# 

**Published:** 1900-01-27

**Authors:** 


					THE HOSPITAL. The standard of highest purity."?Lancet. January 27th, 1900,
CADBURY'S rf H CADBURY'S
GOOOA % MM .JlBU GOOOA
ABSOLUTELY PURE. ^ "O DRUGS OR ALKALIES USED.
h
rui-ASConr Western Infi'rmar
No. 696/ iVol. XXVII. [?w^] Saturday,
Jan. 27, 1900.
[SuhscriptionTenris.J pRICE TWOPENCE.

				

